# Burden of Dementia in Europe, 1990–2023: a systematic analysis from the Global Burden of Disease Study 2023

**DOI:** 10.1016/j.lanepe.2026.101796

**Published:** 2026-07-31

**Authors:** Daniele Urso, Emma Nichols, Stefano Giannoni-Luza, Carol Brayne, Nicolas Ray, Giancarlo Logroscino

**Affiliations:** a Center for Neurodegenerative Diseases and the Aging Brain, University of Bari ‘Aldo Moro’, “Pia Fondazione Cardinale G. Panico”, Tricase, Lecce, Italy; b GeoHealth group, Institute of Global Health, Faculty of Medicine, University of Geneva, Geneva, Switzerland; c Leonard Davis School of Gerontology, University of Southern California, Los Angeles, CA, USA; d Center for Economic and Social Research, University of Southern California, Los Angeles, CA, USA; e Department of Translational Biomedicine and Neuroscience (DiBraiN), University of Bari ‘Aldo Moro’, Bari, Italy; f Cambridge Public Health, University of Cambridge, Cambridge, UK

**Keywords:** Dementia, GBD, Europe, European Union, Global burden of disease, Prevalence, DALYs, Mortality, Risk factors

## Abstract

**Background:**

Dementia is a leading cause of disability and mortality in Europe, yet no recent harmonised assessment has described its impact across the European Union (EU-27) and the WHO European Region.

**Methods:**

We used data from the Global Burden of Disease Study 2023 (GBD 2023) to estimate prevalence, mortality, and disability-adjusted life years (DALYs) for dementia from 1990 to 2023. Estimates were produced for the EU-27 and the WHO European Region, by age and sex. Non-fatal outcomes were modelled using DisMod-MR 2.1, and dementia-attributable mortality was estimated using an excess-mortality framework. We also quantified DALYs attributable to six modifiable risk factors.

**Findings:**

In 2023, 7.76 million people (95% UI 6.69–8.72) were living with dementia in the EU-27 and 12.28 million (10.44–13.90) in the WHO European Region, representing ∼90% increases since 1990 despite modest declines in age-standardised prevalence. Prevalence was nearly twice as high in women as in men. Dementia rose from the eighth to the third leading cause of death in the EU-27. Dementia accounted for 5.49 million DALYs (2.44–11.41) in the EU-27. An estimated 41% (24.4–57.4) of dementia DALYs in the EU-27 were attributable to modifiable risk factors, particularly ambient particulate matter pollution, high fasting plasma glucose, and high body-mass index, which are disproportionately concentrated in socioeconomically disadvantaged populations.

**Interpretation:**

Despite modest declines in age-standardised rates, the absolute burden of dementia in Europe continues to rise, driven by population ageing. The substantial contribution of modifiable risk factors highlights major opportunities for prevention. Robust, country-specific estimates are essential to guide integrated strategies combining prevention and care planning.

**Funding:**

10.13039/501100009886Regione Puglia and CNR for Tecnopolo per la Medicina di Precisione; Regione Puglia for the national “Fund for Alzheimer's and Dementia 2021–2023” (Piano Regionale Demenze 2021/2023).

**Research in context**

**Evidence before this study**

We searched PubMed from database inception to Sept 15, 2025, for articles on the prevalence, mortality, and overall impact of all-cause dementia in Europe, using the search terms (“Prevalence” OR “Mortality” OR “Burden of disease”) AND (“Meta-analysis” OR “Systematic Review”) AND (“Dementia”) AND (“Europe”), with no language or time restrictions. Although several studies have examined dementia frequency and outcomes in Europe, we identified only a small number of systematic reviews and meta-analyses, many of which were limited to specific dementia subtypes, restricted age ranges, or based on older data. The *Dementia in Europe Yearbook 2019* provided prevalence estimates across European countries, drawing primarily on national survey and cohort data. However, it was restricted to prevalence, did not capture individuals younger than 60 years, and did not present comprehensive estimates of mortality or disability burden. The Global Burden of Diseases, Injuries, and Risk Factors (GBD) Study has regularly reported dementia estimates for 204 countries and territories, including European countries, but no dedicated publication has systematically described GBD dementia findings in Europe. Updated and detailed estimates are particularly relevant given the recent approval of disease-targeted therapies for early symptomatic Alzheimer's disease and the urgent need for planning of health and social care services.

**Added value of this study**

This study provides the most recent and comprehensive assessment of dementia burden in Europe, presenting updated GBD 2023 estimates for prevalence, mortality, and disability-adjusted life years (DALYs) at both the country and subregional level (Western, Central, and Eastern Europe). By using the GBD framework, this study goes beyond a conventional systematic review and meta-analysis by harmonising heterogeneous data sources and generating internally consistent estimates across outcomes, countries, age groups, sex, and calendar years. We provide detailed age- and sex-specific estimates in 5-year age bands from age 40 years onwards, highlighting the steep increase in dementia burden at older ages and the disproportionate impact on women. We also present temporal trends from 1990 to 2023, enabling assessment of long-term changes. Furthermore, we report on the contribution of modifiable risk factors, identifying ambient air pollution as the leading contributor to dementia DALYs in Europe among the risk factors currently assessed within the GBD framework.

**Implications of all the available evidence**

The impact of dementia in Europe has nearly doubled over the past three decades, largely driven by population growth and ageing. Robust and up-to-date epidemiological estimates are essential to inform prevention policies, anticipate care needs, and address health inequalities. In parallel, emerging disease-targeted therapies for early symptomatic Alzheimer's disease further increase the need for accurate population-level, and where possible subtype- and stage-specific, data to support informed planning. At the same time, addressing modifiable risk factors, particularly air pollution but also smoking, obesity, and metabolic risks, remains a critical public health priority, especially given evidence from multimodal interventions indicating that modifying risk factor profiles can delay cognitive decline and reduce dementia incidence. The estimates presented here provide a foundation for policymakers to design strategies that integrate possible new treatment opportunities with prevention efforts, while helping to identify underserved and socioeconomically disadvantaged populations who may remain underdiagnosed or underrepresented in routine data systems, and to ensure equitable and sustainable support for people living with dementia and their caregivers across Europe.

## Introduction

Dementia is a leading cause of disability and mortality, with profound consequences for patients, families, and health systems worldwide.^1^ In 2019, an estimated 57.4 million people were living with dementia globally, and this number is projected to nearly triple to 152.8 million by 2050.^2^ Although age-standardised prevalence has remained largely stable or declined slightly in several high-income countries,^3^^,^^4^ absolute numbers continue to rise, driven primarily by population growth and demographic ageing.^2^

Europe is the oldest continent demographically, with more than one in five individuals already aged 65 years or older, and projections indicating that older adults will comprise nearly a third of the population by 2050.^5^ This age structure makes Europe particularly vulnerable to age-related neurological disorders such as dementia. Although several studies have examined dementia incidence and prevalence in Europe, only a limited number of systematic reviews and meta-analyses exist, many restricted to specific dementia subtypes,^6^ narrow age ranges,^7^ or are based on older data.^6^^,^^7^ The *Dementia in Europe Yearbook 2019*^8^ provided valuable prevalence estimates across European countries, but was limited to prevalence, excluded individuals younger than 60 years, and did not present comprehensive estimates of mortality or disability burden. The Global Burden of Disease (GBD) study has previously produced comparable dementia estimates at global, regional, and national levels, offering a framework to capture prevalence, mortality, and disability-adjusted life years (DALYs) using standardised methods across geographies.^9^ Earlier GBD iterations have been widely used to benchmark the global impact of dementia and to inform health policy.^2^ The most recent GBD update provides an opportunity to generate the most comprehensive and up-to-date estimates of dementia burden in Europe, allowing for consistent comparisons across countries and over time.

Accurate estimates of dementia prevalence and burden are essential not only to plan medical care but also to inform prevention strategies.^10^ Evidence has increasingly established that dementia is, to a large extent, preventable,^11^ with nearly half of cases worldwide attributable to modifiable risk factors operating across the life course, including less education, hearing loss, hypertension, diabetes, smoking, obesity, physical inactivity, depression, social isolation, air pollution, traumatic brain injury, and more recently vision loss and high cholesterol. Intervening on these factors through policies and health programmes can delay onset, reduce incidence, and compress morbidity, thereby extending healthy life expectancy.^10^ At the same time, Alzheimer's disease, the most common dementia subtype, accounting for 60–70% of cases,^12^ has entered a new therapeutic era with the approval of anti-β amyloid monoclonal antibodies by both the FDA and EMA. These treatments have downsides, given modest effects, potentially harmful side-effects, requiring intensive resources allocation.^13^ However, they also mark the first disease-targeted therapies in Europe for selected patients with early symptomatic Alzheimer's disease and highlight the urgency of accurate, country-specific estimates to guide eligibility,^14^ infrastructure needs, and economic planning.^15^ Together, prevention and treatment perspectives underscore the importance of robust epidemiological data to inform both population-level interventions and health system preparedness.

Therefore, we aimed to estimate dementia prevalence, mortality, and disability-adjusted life years (DALYs) as both absolute numbers and age-standardised rates in the European Union (EU-27), stratified by age, sex, year, and location for the period 1990–2023, and to compare these estimates with those for the broader WHO European Region and its subregions. In addition, we assessed the contribution of modifiable risk factors included in the GBD 2023 framework, thereby incorporating newly recognised exposures, to provide a comprehensive assessment of dementia burden and its drivers in Europe.

## Methods

### Study overview

The Global Burden of Diseases, Injuries, and Risk Factors Study 2023 (GBD 2023) provides annual, comparable estimates for 371 causes and 88 risk factors across 204 countries and territories, covering the period 1990–2023. Each new release incorporates additional data and refinements to modelling approaches and replaces all previous rounds, thereby ensuring that the entire time series reflects the most updated information. GBD adheres to the Guidelines for Accurate and Transparent Health Estimates Reporting (GATHER). An international network of more than 20,000 collaborators from over 160 countries and territories contributed to data identification, review, and assessment. This study focuses on Alzheimer's disease and other dementias (hereafter referred to as dementia), using estimates produced by the GBD 2023 Causes of Death Collaborators^16^ and the GBD 2023 Disease, Injury, and Risk Factor Collaborators,^17^ and made publicly available through the GBD Results Tool (https://vizhub.healthdata.org/gbd-results/). Dementia was defined according to reference case definitions from the *Diagnostic and Statistical Manual of Mental Disorders* (DSM-III, DSM-IV, DSM-5) and the *International Classification of Diseases* (ICD-8, ICD-9, ICD-10). Relevant ICD-9 codes included 290–290.9, 294.1–294.9, and 331–331.2; relevant ICD-10 codes included F00–F02.0, F02.8–F03.9, G30–G31.1, and G31.8–G31.9. To improve comparability across studies and over time, GBD uses a reference case definition for dementia based on epidemiological assessment with objective cognitive testing and classification according to DSM and ICD criteria, and adjusts alternative diagnostic approaches to this reference before modelling.^16^^,^^17^

### Data sources and processing

Data sources used to produce GBD 2023 estimates are listed in the GBD 2023 Sources Tool (https://ghdx.healthdata.org/gbd-2023/sources). For dementia, non-fatal estimates were informed by prevalence and incidence data from population-based cross-sectional and longitudinal studies, surveys, and selected administrative data sources such as claims data identified through systematic review and synthesised within the DisMod-MR 2.1 framework. GBD 2023 additionally incorporated sex-specific reference-case prevalence and incidence data identified through an updated systematic review of population-based studies through February, 2023, and results from 18 studies participating in the Cohort Studies of Memory in an International Consortium (COSMIC).^17^ Because dementia mortality is particularly sensitive to temporal and geographic variation in death certification and coding practices, fatal estimation did not rely directly on raw vital registration trends; instead, it used an attributable-risk framework informed by systematic-review evidence on excess mortality among individuals with dementia and linked hospitalisation–mortality data.^16^ These inputs include both population-based epidemiological studies and other data sources, such as surveys and administrative data, and their availability varies across European countries. To adjust heterogeneous, non-reference data sources and account for differences we first cross-walked data using a matched pairs meta-regression analysis.^18^ Meta-regression results were used to systematically adjust non-reference data and account for inconsistencies. Using the GBD 2023 Sources Tool, we summarised and visualised country-level data availability in Europe for Alzheimer's disease and other dementias (non-fatal outcomes and cause of death inputs), to contextualise the strength and heterogeneity of the evidence underlying GBD 2023 estimates (Appendix Figure S1).

### Modelling of non-fatal and fatal dementia estimates

Prior to non-fatal modelling, non-fatal outcomes (incidence, prevalence, and years lived with disability [YLDs]) were modelled using DisMod-MR 2.1, a Bayesian meta-regression framework that integrates heterogeneous data sources and enforces internal consistency across key epidemiological parameters, including incidence, prevalence, and mortality. To prevent double counting across GBD causes, dementia prevalence estimates were adjusted to exclude cases attributable to other conditions that can cause dementia and are modelled separately in GBD, including stroke, Parkinson's disease, traumatic brain injury, and Down syndrome. This adjustment was based on age-specific relative risks derived from epidemiological studies and systematic reviews,^19^ combined with estimates of the prevalence of these conditions. YLDs were estimated by multiplying prevalence by the disability weights assigned to different severity levels of dementia, reflecting the magnitude of health loss associated with each state. Severity splits and disability weights were derived from population surveys and standardised across locations, as described in the GBD 2023 capstone publications.^16^^,^^17^ Unlike most causes in GBD, non-fatal outcomes for dementia are modelled jointly with mortality to account for known inconsistencies between prevalence data and cause-of-death coding over time.^17^ Accordingly, long-term trends should be interpreted as harmonised modelled estimates generated under a standardised reference-case framework, rather than as direct comparisons of studies using unadjusted diagnostic classifications across time. This is achieved by applying crosswalk adjustments that map alternative case definitions and ascertainment methods to a common reference-case definition prior to modelling. Dementia mortality estimates represent mortality attributable to dementia as an underlying cause of death. Estimates were derived using an attributable-risk framework based on excess mortality among individuals with dementia. This approach does not rely directly on whether dementia is recorded as the underlying or a contributory cause of death, and is intended to correct for known biases in death certification and coding practices across locations and over time.^16^ Years of life lost (YLLs) were derived by multiplying deaths by the standard life expectancy at age of death, and DALYs were calculated as the sum of YLLs and YLDs. A full description of input data, modelling procedures, and disability weights used to estimate severity distributions for YLDs is provided in the GBD 2023 capstone papers and appendices.^16^^,^^17^ In accordance with the natural history of dementia,^2^ GBD assumes zero incidence and zero remission before age 40 years.

### Risk-attributable burden

GBD quantifies attributable burden using a comparative risk assessment framework. This framework enables the quantification of potentially preventable dementia burden and facilitates comparison of modifiable risk factors to inform public health prioritisation. In this framework, attributable DALYs are estimated by comparing observed exposure distributions with a counterfactual exposure distribution, the theoretical minimum risk exposure level (TMREL), defined as the level or distribution of exposure that minimises population risk. Population attributable fractions (PAFs) therefore represent the proportion of dementia DALYs expected to be reduced if exposure were shifted to the TMREL. For dementia, six modifiable risk factors were included in the GBD 2023 comparative risk assessment framework based on the strength of the existing evidence linking these risks with dementia: ambient particulate matter pollution, high alcohol use, smoking, high body-mass index, household air pollution from solid fuels, and high fasting plasma glucose. Exposure was estimated separately by location, age, sex, and year using risk-specific modelling approaches and data sources that vary by risk, including surveys, measurement data, administrative sources, and, for environmental exposures, satellite and geospatial inputs. Detailed definitions and modelling procedures for each risk are provided in the GBD 2023 risk factor Appendix.^17^

### Statistical analysis

For the present analysis, we used dementia estimates for the 27 European Union (EU-27) Member States, the WHO European Region (53 countries), and the GBD European subregions (Western, Central, and Eastern Europe). Outcomes were presented as counts and age-standardised rates per 100,000 population. Both absolute counts and age-standardised rates were reported, together with percentage changes between 1990 and 2023 to disentangle demographic effects from changes in underlying disease risk. To explore the potential role of socioeconomic inequalities in dementia burden, we examined the association between the Socio-demographic Index (SDI), a composite measure of country-level socioeconomic development based on income, educational attainment, and fertility, and age-standardised DALY rates for dementia across European countries in 2023.^17^ Correlations between SDI and age-standardised DALY rates were assessed using Spearman's rank correlation coefficients for EU-27 countries and for countries in the WHO European Region. To quantify the contribution of demographic and epidemiological changes to the increase in dementia burden, we performed a symmetric decomposition analysis^2^ of the change in total dementia DALYs between 1990 and 2023, partitioning the difference into contributions from population growth, population ageing, and changes in age-specific DALY rates. The decomposition used age-specific DALY counts and population counts (both sexes combined) and was done separately for the EU-27 and the WHO European Region.

### Calculating uncertainty

Uncertainty was introduced throughout the estimation process. Mean estimates for all other metrics reported represent the mean value across 250 draws from the estimate's distribution, with 95% uncertainty intervals (UIs) calculated as the 2.5 and 97.5 percentile values across the draws. To reduce computing power and time across the estimation process, the number of draws was reduced from 500 in previous GBD iterations to 250 for GBD 2023. Simulations confirmed that estimates and uncertainty were not impacted by this reduction (see GBD 2023 Disease and Injury and Risk Factors Collaborators^17^ for more details).

### GBD researching and reporting practices

GBD 2023 complies with GATHER guidelines; the GATHER checklist for this study is available in the Appendix Table S1. The University of Washington Institutional Review Board approved the GBD study (STUDY00009060) and GBD 2023 statistical code is publicly available online (http://ghdx.healthdata.org/gbd-2023/code).

### Role of the funding source

The funders of this study had no role in study design, data collection, data analysis, data interpretation, or the writing of the report.

## Results

In 2023, the total population in the EU-27 was 453.8 million and the population of WHO European region was 948.5 million. The total number of people living with dementia in the EU-27 was 7.76 million (95% UI 6.69–8.72), corresponding to an age-standardised prevalence of 651.3 per 100,000 population (562.0–732.5, Table 1). Compared to 1990, this represents an increase of 88.5% (82.9–94.6) in the number of cases but a reduction of 1.7% (−4.2 to 0.8) in age-standardised prevalence. In the WHO European Region, dementia prevalence reached 12.28 million cases (10.44–13.90) with an age-standardised prevalence of 634.1 per 100,000 (541.2–714.4). Between 1990 and 2023, counts increased by 80.9% (76.6–84.9), while age-standardised prevalence showed a reduction of 1.7% (−3.5 to 0.0).

**Table 1:** Prevalence, DALY and deaths for dementia in 2023, and percentage change of age-standardized rates and counts by location in Europe, 1990–2023.

Area	Counts 2023 (95% UI)	Percentage change in counts, 1990–2023 (95% UI)	Age-standardised rate 2023 per 100,000 (95% UI)	Percentage change in age-standardised rate, 1990–2023 (95% UI)
Prevalence				
European Union	7,760,423 (6,691,560–8,717,760)	88.5% (82.9–94.6)	651.3 (562.0–732.5)	−1.7% (−4.2 to 0.8)
European Region	12,276,497 (10,437,880–13,895,747)	80.9% (76.6–84.9)	634.1 (541.2–714.4)	−1.7% (−3.5 to −0.0)
Western Europe	7,858,887 (6,760,781–8,847,355)	86.3% (80.9–91.7)	663.7 (571.1–746.6)	−2.5% (−4.9 to −0.1)
Central Europe	1,336,198 (1,119,551–1,530,429)	77.7% (72.8–82.4)	555.1 (469.7–632.9)	−1.6% (−3.1 to −0.3)
Eastern Europe	2,109,392 (1,761,704–2,441,423)	45.4% (42.1–49.1)	573.1 (484.6–662.1)	−2.3% (−3.6 to −1.2)
Austria	136,123 (112,202–157,144)	66.0% (58.7–73.6)	607.7 (507.7–700.4)	−5.2% (−9.3 to −1.4)
Belgium	194,677 (162,400–223,987)	60.3% (50.1–70.5)	680.3 (573.8–777.3)	−8.6% (−14.0 to −3.8)
Bulgaria	88,565 (73,783–102,498)	46.5% (39.2–53.3)	550.0 (465.0–633.8)	0.4% (−3.7–4.1)
Croatia	54,618 (45,939–63,064)	67.1% (58.6–75.2)	560.6 (477.7–642.4)	−2.5% (−6.2–1.3)
Cyprus	12,417 (10,311–14,322)	139.9% (130.0–150.1)	626.5 (522.2–720.6)	−1.8% (−5.4–1.8)
Czechia	131,360 (109,895–152,185)	84.1% (75.3–94.2)	548.7 (466.0–626.7)	−0.5% (−4.6–4.1)
Denmark	67,621 (57,474–77,288)	38.5% (31.1–45.7)	473.7 (404.5–536.3)	−14.4% (−19.1 to −10.8)
Estonia	18,563 (15,593–21,434)	63.9% (56.1–72.2)	559.5 (473.7–638.6)	−2.1% (−6.3–1.9)
Finland	97,288 (80,563–111,407)	104.4% (90.9–116.7)	618.8 (514.9–706.9)	−7.6% (−13.1 to −2.8)
France	893,571 (778,532–1,006,963)	76.3% (68.2–84.7)	498.9 (439.1–566.0)	−4.7% (−9.1 to −0.4)
Germany	2,117,751 (1,854,183–2,371,713)	85.5% (72.4–98.8)	852.5 (744.7–958.0)	2.2% (−4.4–9.2)
Greece	252,013 (211,401–292,857)	113.8% (98.9–131.7)	777.8 (660.5–896.7)	−0.9% (−6.8–6.3)
Hungary	118,367 (100,484–134,458)	54.4% (47.5–61.3)	555.8 (475.5–629.5)	1.1% (−3.0–5.3)
Ireland	56,191 (46,487–64,301)	115.8% (106.3–124.4)	605.7 (503.8–690.2)	−5.4% (−8.6 to −2.3)
Italy	1,427,709 (1,186,279–1,656,291)	122.2% (110.4–135.2)	760.1 (638.7–879.6)	8.9% (4.3–13.5)
Latvia	27,171 (22,934–31,557)	35.0% (28.8–40.8)	564.6 (478.5–649.2)	−0.4% (−4.2–3.0)
Lithuania	38,173 (32,284–44,220)	52.9% (46.3–60.0)	556.8 (473.9–639.2)	−1.5% (−5.6–2.3)
Luxembourg	5358 (4481–6259)	83.8% (62.1–101.7)	462.0 (387.4–536.5)	−12.1% (−22.0 to −4.7)
Malta	7124 (5972–8197)	184.6% (170.6–198.4)	616.9 (521.6–703.2)	−4.4% (−8.3 to −0.7)
Netherlands	290,303 (241,496–329,863)	112.4% (99.6–128.6)	684.8 (576.9–774.5)	2.7% (−3.1–10.0)
Poland	447,037 (369,100–517,934)	86.6% (80.6–93.1)	559.1 (462.9–646.2)	−5.3% (−7.0 to −4.0)
Portugal	173,437 (144,813–201,507)	124.5% (110.9–139.3)	550.8 (466.5–632.0)	−3.6% (−8.4–0.6)
Romania	223,004 (187,699–257,604)	66.4% (58.8–75.3)	556.5 (474.5–638.4)	0.2% (−3.9–4.1)
Slovakia	54,053 (45,996–61,726)	81.6% (73.8–89.1)	547.0 (469.0–625.9)	1.0% (−2.9–4.8)
Slovenia	28,987 (24,240–33,335)	130.8% (121.1–142.2)	550.9 (467.8–631.4)	−2.3% (−5.6–1.4)
Spain	644,939 (543,484–741,311)	85.5% (70.8–101.3)	515.8 (437.9–589.4)	−17.9% (−24.5 to −11.5)
Sweden	154,003 (129,344–177,609)	58.2% (47.8–72.1)	565.5 (479.3–642.2)	−2.1% (−8.4–5.8)
United Kingdom	1,037,147 (859,980–1,200,624)	58.1% (53.3–62.2)	639.2 (532.0–735.3)	−5.1% (−7.2 to −3.1)
Switzerland	141,371 (118,331–163,854)	81.1% (73.4–89.7)	632.7 (534.3–728.3)	−6.2% (−9.8 to −2.6)
Norway	86,256 (71,078–99,999)	51.3% (44.8–56.6)	725.0 (599.2–837.4)	−4.9% (−8.6 to −2.7)
Iceland	4355 (3613–4969)	100.8% (84.2–114.3)	657.7 (545.5–750.2)	−10.0% (−18.1 to −3.7)
DALYs				
European Union	5,493,619 (2,435,786–11,405,129)	98.9% (90.2–107.5)	446.0 (197.8–909.0)	−2.5% (−5.7–0.6)
European Region	8,564,505 (3,809,663–17,708,944)	94.3% (86.3–101.3)	435.3 (193.4–896.8)	−0.2% (−3.1–2.4)
Western Europe	5,566,991 (2,478,575–11,517,987)	97.2% (88.6–105.4)	451.8 (201.3–916.7)	−2.8% (−6.1–0.1)
Central Europe	896,522 (396,286–1,920,968)	88.0% (80.6–95.8)	374.6 (165.8–803.0)	−1.6% (−3.8–0.5)
Eastern Europe	1,431,066 (636,212–3,014,607)	62.7% (54.8–71.6)	394.1 (174.5–835.7)	4.1% (0.4–8.5)
Austria	97,054 (43,110–203,243)	77.1% (68.2–86.4)	422.3 (187.6–871.3)	−4.5% (−8.7 to −0.6)
Belgium	140,914 (63,710–291,109)	73.9% (63.5–84.3)	471.9 (213.4–961.4)	−7.1% (−12.0 to −2.8)
Bulgaria	54,637 (24,436–113,717)	44.6% (37.1–53.7)	352.5 (157.6–734.7)	−4.4% (−9.0–1.1)
Croatia	36,631 (16,187–78,014)	80.5% (68.3–92.6)	377.4 (166.6–790.2)	−1.9% (−6.5–2.6)
Cyprus	8879 (3916–18,957)	153.3% (120.6–188.7)	470.7 (204.5–1008.8)	−0.0% (−12.7–14.8)
Czechia	84,717 (37,911–179,068)	86.9% (77.5–97.5)	360.0 (160.6–755.1)	−3.3% (−8.3–2.0)
Denmark	56,271 (22,910–122,872)	66.6% (52.7–76.1)	388.1 (158.6–841.7)	−1.0% (−7.9–4.0)
Estonia	13,653 (5929–28,631)	94.7% (79.8–110.0)	398.7 (174.2–833.2)	7.4% (1.4–14.8)
Finland	74,368 (32,239–155,754)	131.1% (115.2–144.8)	460.2 (200.3–955.8)	−2.8% (−8.2–2.1)
France	716,259 (297,428–1,532,739)	91.7% (79.7–102.8)	379.2 (159.0–795.3)	−0.8% (−6.1–4.5)
Germany	1,334,097 (607,726–2,659,026)	89.0% (75.1–102.5)	515.9 (237.1–1016.2)	−1.5% (−8.5–5.2)
Greece	151,094 (71,540–313,925)	123.8% (111.0–137.8)	458.1 (219.2–941.6)	−3.2% (−7.2–2.4)
Hungary	79,900 (35,351–169,239)	62.9% (55.8–70.5)	376.0 (166.8–786.9)	1.7% (−2.1–6.4)
Ireland	39,893 (17,611–82,676)	135.6% (121.5–149.4)	429.3 (189.4–888.3)	−2.2% (−7.6–4.0)
Italy	1,030,054 (472,539–2,091,364)	118.0% (106.2–137.7)	523.1 (239.0–1054.5)	−2.0% (−7.1–6.0)
Latvia	18,919 (8394–40,378)	44.9% (35.1–53.8)	385.9 (170.8–806.5)	0.6% (−4.7–5.8)
Lithuania	26,400 (11,452–56,312)	67.2% (56.0–77.8)	380.1 (164.3–810.7)	1.7% (−3.5–7.3)
Luxembourg	3656 (1681–7777)	112.1% (92.5–128.6)	311.1 (143.9–662.6)	−4.6% (−11.3–1.4)
Malta	4882 (2196–9798)	202.1% (185.5–224.1)	428.2 (192.5–859.0)	−2.9% (−8.5–3.2)
Netherlands	204,628 (87,919–428,323)	116.6% (104.6–127.6)	476.1 (205.9–985.7)	0.6% (−4.7–5.8)
Poland	308,637 (135,380–657,507)	102.8% (92.6–112.2)	380.4 (168.6–807.4)	−3.8% (−6.5 to −0.8)
Portugal	141,210 (60,699–301,941)	155.4% (136.5–174.3)	430.2 (184.1–905.1)	−0.4% (−6.2–5.3)
Romania	143,960 (65,136–304,075)	72.9% (63.4–83.0)	362.5 (163.7–769.2)	−2.5% (−6.2–2.1)
Slovakia	35,911 (16,019–76,801)	87.2% (74.5–100.6)	369.5 (164.6–789.9)	0.9% (−6.6–9.1)
Slovenia	21,098 (9116–45,535)	163.1% (143.9–182.0)	391.5 (168.8–841.6)	2.6% (−1.9–7.6)
Spain	550,525 (229,942–1,166,768)	123.5% (107.4–137.7)	412.1 (174.6–854.2)	−10.0% (−14.4 to −5.1)
Sweden	115,373 (48,826–242,624)	65.2% (56.5–74.1)	412.3 (176.9–849.1)	−2.0% (−6.4–2.9)
United Kingdom	676,490 (312,719–1,405,500)	66.6% (60.6–72.4)	406.5 (189.7–837.5)	−5.3% (−7.9 to −2.7)
Switzerland	100,691 (45,313–206,214)	100.0% (90.5–112.4)	430.8 (194.5–869.3)	−2.3% (−6.1–2.1)
Norway	56,958 (25,893–117,469)	59.2% (50.1–66.9)	467.8 (214.0–955.0)	−4.0% (−9.4 to −0.0)
Iceland	2940 (1324–6084)	120.9% (104.6–134.0)	436.7 (197.0–898.3)	−3.7% (−11.2–1.2)
Deaths				
European Union	343,526 (86,199–841,112)	124.2% (111.0–142.3)	26.2 (6.6–64.2)	−1.8% (−5.9–4.7)
European Region	514,969 (127,525–1,261,212)	118.7% (108.7–134.9)	25.4 (6.3–62.3)	1.2% (−3.0–6.7)
Western Europe	352,229 (88,995–858,048)	121.2% (108.4–138.9)	26.6 (6.7–64.7)	−1.9% (−6.0–4.5)
Central Europe	49,767 (12,012–128,772)	114.9% (104.5–131.0)	21.1 (5.1–54.8)	−0.9% (−4.0–3.1)
Eastern Europe	77,689 (18,993–195,129)	83.8% (72.9–101.3)	22.4 (5.4–56.6)	7.4% (2.6–15.7)
Austria	5998 (1479–14,826)	97.1% (84.9–115.6)	25.0 (6.1–61.8)	−2.9% (−8.2–4.1)
Belgium	8986 (2246–21,980)	98.2% (82.2–119.7)	28.0 (6.9–68.3)	−5.2% (−11.3–3.2)
Bulgaria	2810 (672–7462)	58.1% (44.8–71.4)	19.4 (4.6–52.0)	−5.8% (−13.2–1.3)
Croatia	2063 (509–5322)	113.3% (98.3–135.3)	21.4 (5.3–55.6)	−0.4% (−7.0–7.1)
Cyprus	522 (136–1291)	173.3% (124.7–242.5)	29.3 (7.5–72.1)	1.4% (−17.4–25.7)
Czechia	4631 (1133–11,918)	106.3% (92.5–125.3)	20.1 (4.9–51.9)	−4.0% (−9.4–4.5)
Denmark	3612 (888–9239)	89.4% (77.2–106.1)	24.3 (6.0–62.1)	6.5% (−0.1–15.5)
Estonia	842 (208–2078)	136.5% (115.7–166.8)	23.3 (5.8–57.8)	13.1% (4.9–24.7)
Finland	4759 (1180–11,593)	169.2% (146.9–202.6)	28.0 (7.0–68.0)	−0.2% (−7.1–9.9)
France	47,732 (11,964–119,424)	109.6% (94.9–131.5)	23.2 (5.7–57.9)	2.0% (−4.9–11.7)
Germany	81,429 (20,923–197,796)	107.6% (87.6–138.8)	29.4 (7.6–71.4)	−2.2% (−10.7–10.9)
Greece	8724 (2133–22,474)	160.6% (141.4–189.2)	24.8 (6.1–64.1)	−4.2% (−9.3–4.7)
Hungary	4480 (1089–11,638)	81.1% (70.8–95.3)	21.1 (5.1–55.1)	1.6% (−3.9–9.2)
Ireland	2399 (594–5892)	166.9% (146.3–199.7)	25.6 (6.3–62.9)	0.5% (−6.8–10.9)
Italy	66,039 (17,095–157,342)	144.5% (128.9–170.5)	30.9 (8.0–73.6)	−4.8% (−9.1–2.3)
Latvia	1117 (271–2948)	63.3% (48.3–83.0)	21.9 (5.4–57.8)	0.7% (−6.9–11.0)
Lithuania	1554 (374–4042)	88.5% (72.7–109.1)	21.7 (5.2–56.5)	2.9% (−4.9–12.2)
Luxembourg	218 (54–566)	150.9% (127.9–181.8)	18.1 (4.5–47.0)	1.2% (−6.8–11.2)
Malta	284 (70–692)	243.8% (216.3–290.3)	25.0 (6.2–60.6)	−1.0% (−9.0–10.8)
Netherlands	12,538 (3093–30,974)	130.9% (114.5–148.8)	28.4 (7.0–70.0)	0.1% (−6.4–7.9)
Poland	17,771 (4418–45,277)	136.5% (120.9–162.5)	21.5 (5.3–54.9)	−2.3% (−6.4–3.8)
Portugal	9304 (2383–22,977)	206.7% (179.1–248.7)	26.3 (6.6–64.8)	0.8% (−6.7–11.0)
Romania	7744 (1784–20,176)	100.7% (87.0–119.8)	19.9 (4.6–52.2)	−3.0% (−9.1–4.5)
Slovakia	1946 (483–5018)	105.4% (86.4–138.7)	20.9 (5.2–53.9)	3.2% (−7.3–21.5)
Slovenia	1280 (326–3257)	219.5% (193.1–257.0)	22.7 (5.8–57.9)	6.3% (−0.8–16.8)
Spain	37,393 (9622–91,328)	172.0% (150.1–203.2)	25.3 (6.5–61.3)	−6.5% (−11.7–1.8)
Sweden	7350 (1848–18,432)	79.9% (67.3–98.2)	24.9 (6.3–61.9)	−0.9% (−6.7–8.2)
United Kingdom	40,685 (9949–101,256)	83.9% (76.9–95.5)	23.1 (5.7–57.4)	−5.3% (−8.4 to −0.7)
Switzerland	6400 (1631–15,384)	130.1% (115.3–158.4)	25.4 (6.4–60.9)	3.2% (−2.5–13.5)
Norway	3423 (845–8600)	75.9% (65.0–93.1)	26.9 (6.6–67.3)	−1.4% (−7.3–6.3)
Iceland	177 (46–441)	145.8% (129.4–166.9)	25.6 (6.6–63.2)	2.3% (−4.6–10.9)

Counts are shown as all-age numbers with 95% uncertainty intervals (UIs). Age-standardised rates are per 100,000 population, with 95% UIs. Percentage changes represent relative differences between 1990 and 2023 estimates. UI, uncertainty interval; DALYs, disability-adjusted life years.

**Fig. 1:**
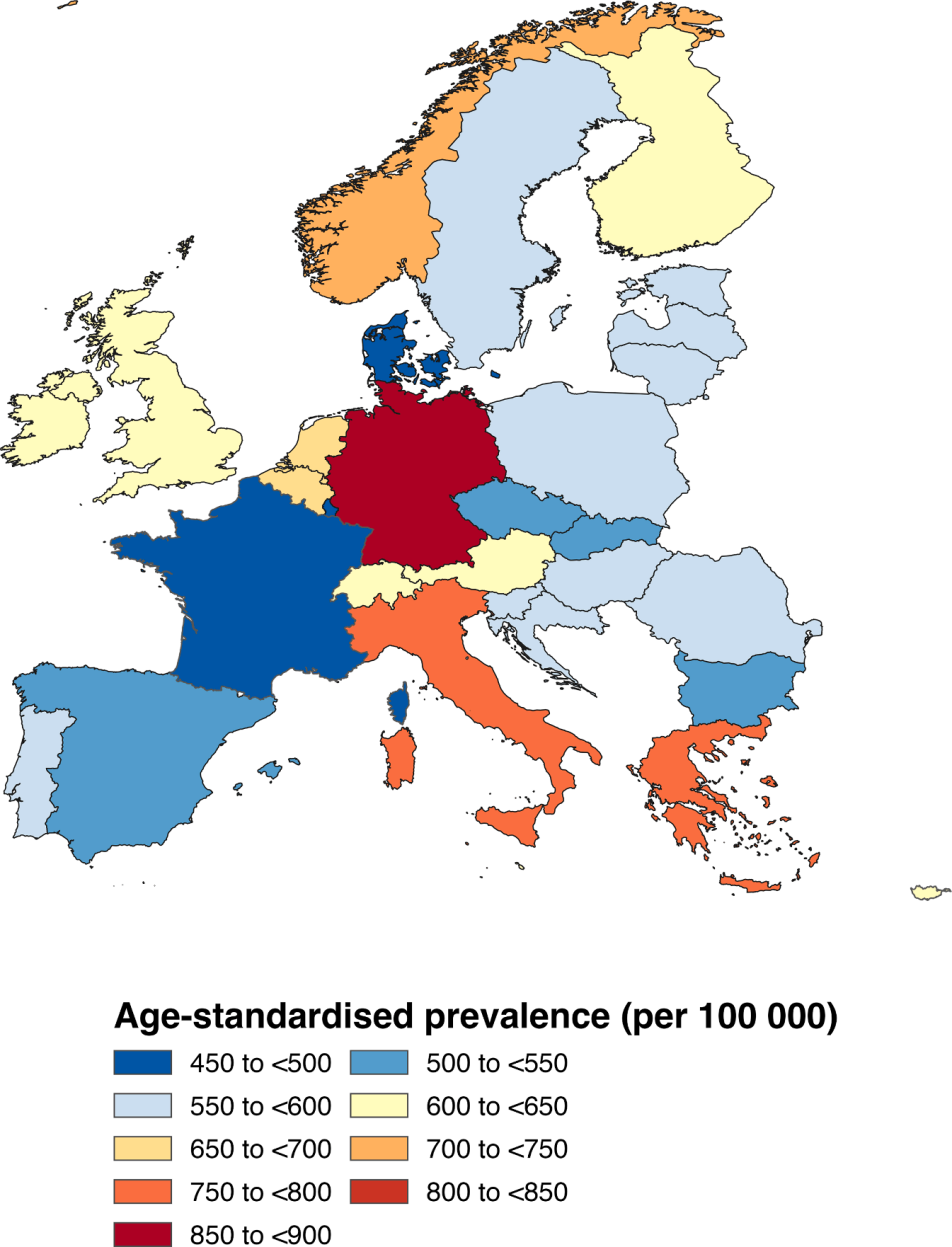
**Age-standardised prevalence rate of dementia per 100,000 population in the European Union, 2023**. Results shown for EU-27 plus the United Kingdom, Switzerland, Norway, and Iceland.

Across GBD subregions, the largest number of cases was observed in Western Europe, with 7.86 million cases (6.76–8.85), followed by Eastern Europe with 2.11 million cases (1.76–2.44), and Central Europe with 1.34 million cases (1.12–1.53). In terms of age-standardised prevalence, Western Europe had the highest rate at 663.7 per 100,000 (571.1–746.6), compared with 573.1 (484.6–662.1) in Eastern Europe and 555.1 (469.7–632.9) in Central Europe, though uncertainty intervals were overlapping. From 1990 to 2023, age-standardised prevalence declined across all subregions, with a reduction of 2.5% (−4.9 to −0.1) in Western Europe, 2.3% (−3.6 to −1.2) in Eastern Europe, and 1.6% (−3.1 to −0.3) in Central Europe. At the country level, age-standardised prevalence varied substantially across the EU-27, with the highest rates observed in Germany, Greece, and Italy, and the lowest in Luxembourg, Denmark, and France (Fig. 1 and Figure S2).

**Fig. 2:**
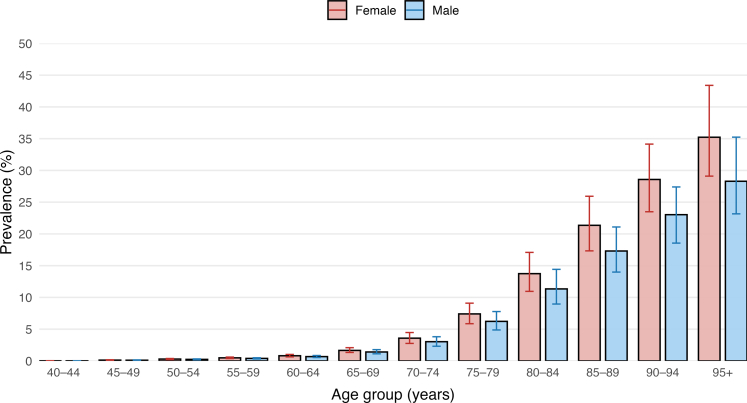
**Age-specific prevalence of dementia in the European Union, 2023, by sex**. Prevalence is expressed as the percentage of the population affected within each 5-year age group. Bars represent point estimates for females and males, with error bars indicating 95% uncertainty intervals.

In 2023, dementia prevalence was consistently higher in women than in men across all age groups in the EU-27 (Fig. 2) and the WHO European region (Figure S3). Before the age of 65 years, dementia prevalence remained below 1% in both sexes, corresponding to about 495,782 people living with dementia in the EU-27. At ages 65–69 years, prevalence was 1.4% (1.1–1.8) in men and 1.7% (1.3–2.1) in women. Among individuals aged 70–74 years, prevalence increased to 3.0% (2.3–3.8) in men and 3.6% (2.8–4.5) in women. By ages 85–89 years, prevalence reached 17.3% (14.0–21.1) in men and 21.4% (17.3–25.9) in women, while in those aged 90–94 years it rose to 29.0% (22.1–36.3) in men and 36.4% (28.4–45.1) in women. Age-standardised prevalence in 2023 was 1.21 times higher in females than in males. In absolute numbers, this corresponded to 5.03 million women (4.35–5.67) and 2.73 million men (2.34–3.10) living with dementia in the EU-27.

In 2023, dementia accounted for an estimated 343,526 deaths (95% UI 86,199–841,112) in the EU-27 and 514,969 deaths (127,525–1,261,212) in the WHO European region, representing increases of 124.2% (111.0–142.3) and 118.7% (108.7–134.9), respectively, since 1990. Despite these large increases in absolute numbers, age-standardised death rates remained relatively stable, with a small, non-significant reduction in the EU-27 (−1.8% [–5.9 to 4.7]) and a slight, non-significant increase in the WHO European region (+1.2% [–3.0 to 6.7]) during 1990–2023. Dementia rose in rank from the eighth leading cause of death in the EU-27 in 1990 to the third in 2023 (Figure S4), and from the seventh in the WHO European region in 1990 to the fourth in 2023 (Figure S5), based on age-standardised death rates. Across European subregions, Western Europe (−26.6 per 100,000, 6.7–64.7) and Central Europe (−21.1, 5.1–54.8) showed modest reductions between 1990 and 2023, whereas Eastern Europe experienced an increase (+7.4% [2.6–15.7]). At the country level, the highest age-standardised death rates in 2023 were observed in Italy (30.9 per 100,000, 8.0–73.6), Germany (29.4, 7.6–71.4), and Finland (28.0, 7.0–68.0), while the lowest were recorded in Luxembourg (18.1, 4.5–47.0), Bulgaria (19.4, 4.6–52.0), and Romania (19.9, 4.6–52.2).

In 2023, dementia accounted for 5.49 million disability-adjusted life years (DALYs; 95% UI 2.44–11.41) in the EU-27 and 8.56 million (3.81–17.71) in the WHO European region. This represented increases of 98.9% (90.2–107.5) and 94.3% (86.3–101.3), respectively, compared with 1990. Despite these increases in absolute numbers, age-standardised DALY rates remained stable or declined only modestly. Decomposition analyses indicated that the increase in dementia DALYs between 1990 and 2023 was driven predominantly by demographic factors, especially population ageing and population growth, whereas changes in age-specific DALY rates slightly offset the increase in both the EU-27 and the WHO European Region (Figure S6). Across European countries, higher socio-demographic development was moderately associated with higher age-standardised DALY rates for dementia. In the WHO European Region, SDI showed a positive correlation with DALYs (Spearman ρ = 0.43, p = 0.001, Figure S7), with similar but slightly weaker patterns in EU-27 countries (Spearman ρ = 0.38, p = 0.048, Figure S8). Dementia ranked as the 9th leading cause of DALYs in the EU-27 and the 16th in the WHO European region in 2023, accounting for 1.61% (95% UI 0.74–3.65) of all DALYs in the EU-27 and 1.14% (0.53–2.60) in the WHO European region.

In 2023, an estimated 2.25 million DALYs (95% UI 0.75–4.96), corresponding to 41.0% (24.4–57.4) of total dementia burden in the EU-27 were attributable to modifiable risk factors analysed as part of GBD. The leading contributor was ambient particulate matter pollution (1.07 million DALYs, 0.16–2.64; 19.6% [4.0–37.9]), followed by high fasting plasma glucose (0.99 million, 0.43–2.05; 18.1% [13.8–23.7]), high body-mass index (0.51 million, −0.17–1.61; 9.8% [–5.2–24.9]), smoking (0.17 million, 0.06–0.35; 3.3% [2.2–4.6]), and household air pollution from solid fuels (0.02 million, 0.001–0.12; 0.3% [0.01–1.6]).

## Discussion

In 2023, an estimated 7.8 million people were living with dementia in the EU-27 and 12.3 million in the WHO European Region, almost double the number of cases observed in 1990. This increase occurred despite a modest 1.7% reduction in age-standardised prevalence rates. Western Europe accounted for the highest number of cases and the greatest age-standardised prevalence. Across both the EU-27 and the WHO European Region, dementia prevalence was consistently higher in women than in men at all ages, and in absolute numbers prevalence was almost twice as high in women as in men. Dementia rose in rank from the eighth leading cause of death in the EU-27 in 1990 to the third in 2023, and from the seventh to the fourth in the WHO European Region over the same period. In terms of overall disease burden, dementia ranked ninth for disability-adjusted life years (DALYs) in the EU-27 and 16th in the WHO European Region in 2023. Ambient particulate matter pollution, high fasting plasma glucose, and high body-mass index emerged as the leading modifiable contributors to dementia burden. These findings highlight the rising toll of dementia in Europe and underscore the urgent need for health systems to prepare for increasing numbers of people affected in the coming decades.

Our estimate of 7.76 million people living with dementia in the EU-27 in 2023 is remarkably close to the figure reported in the *Dementia in Europe Yearbook 2019*,^8^ which corresponded to 7.85 million when recalculated for the same region. This similarity supports the robustness of our estimates, although important methodological differences exist. The Yearbook was restricted to prevalence, excluded individuals younger than 60 years, and did not provide comprehensive information on mortality or disability burden. Previous systematic reviews and meta-analyses in Europe have also been limited,^6^^,^^7^ focussing on specific dementia subtypes, or omitting younger age groups altogether. Yet young-onset dementia, defined as symptom onset before age 65 years, carries distinct implications, affecting family life, occupational functioning, and caregiving needs, and is often complicated by misdiagnosis and delayed access to appropriate care.^20^ Although estimates in this age group are based on a relatively limited number of population-based studies and should therefore be interpreted with caution, our results suggest that nearly half a million people in the EU-27 live with young-onset dementia, highlighting an under-recognised component of the overall impact with substantial and specific clinical, social and economic consequences.

The population structure of the EU-27 and Western Europe is older than that of Central and Eastern Europe, making these regions particularly vulnerable to the growing impact of age-related neurological disorders such as dementia. The steady rise in the number of people living with dementias since 1990 reflects demographic shifts towards greater longevity and declining birth rates. Importantly, population-based evidence indicates that in the oldest age groups dementia at death is the norm rather than the exception. A seminal population-based study showed that more than half of individuals dying after age 95 had dementia, rising to nearly 80% when moderate or severe cognitive impairment was considered.^21^ In this context, the rising prevalence and mortality ranking of dementia largely reflect survival into very old age. This interpretation was supported by decomposition analyses showing that the increase in dementia DALYs between 1990 and 2023 was driven predominantly by demographic change: in the EU-27, population ageing accounted for 55.2% of the increase and population growth for 48.7%, whereas changes in age-specific DALY rates made a small negative contribution (−3.9%); in the WHO European Region, the corresponding contributions were 45.4%, 55.0%, and −0.4%, respectively. Although the absolute number of individuals with dementia continues to increase, our finding of a slight but significant decline in age-specific prevalence is consistent with repeated reports that age-specific incidence of all-cause dementia is falling in high-income countries.^10^ Longitudinal cohort studies from Europe, the United States, and England have documented comparable declines across successive birth cohorts, suggesting a birth cohort effect.^3^^,^^4^ This decline is likely driven by population-level improvements in educational attainment, better prevention and management of cardiovascular and metabolic risk factors, and broader socioeconomic gains.^22^^,^^23^ Collectively, these observations indicate that dementia prevention is not only feasible but already occurring in high-income settings, underscoring the importance of sustained investment in public health and primary prevention strategies.^10^

Our exploratory analyses also suggest a socioeconomic gradient in the measured burden of dementia at the country level across Europe. Countries with higher socio-demographic development tended to have higher age-standardised DALY rates. This finding should not be interpreted as indicating a higher biological risk of dementia in more developed settings, but likely reflects differences in diagnostic capacity, survival, and case ascertainment. In countries with lower socioeconomic development, dementia may be underdiagnosed or underreported, particularly in rural areas or health systems with limited access to specialist care. These patterns highlight that observed cross-country differences in dementia burden partly reflect differences in health system capacity and surveillance infrastructure, and reinforce the importance of strengthening dementia recognition and reporting in underserved populations.

Dementia is increasingly recognised as preventable. The 2024 Lancet Commission^11^ estimated that addressing a suite of modifiable exposures across the life course could theoretically prevent nearly half of cases globally if observed associations were assumed causal, underscoring the centrality of prevention alongside care and emerging disease-targeted therapies. Whereas population attributable fractions (PAFs) quantify the maximum proportion of cases linked to each risk factor,^11^ potential impact fractions (PIFs) are more policy-oriented because they estimate reductions in prevalence under realistic risk-reduction scenarios.^24^ Recent modelling suggests that even a 15–25% proportional reduction in modifiable risk factors could produce measurable declines in dementia prevalence over 10–30 years,^24^ supporting the case for prevention on timescales relevant to health systems. Translating these proportions into the number of preventable cases requires accurate country-specific prevalence estimates, such as those provided by this analysis.

In our analyses, ambient particulate matter pollution, high fasting plasma glucose, and high body-mass index were the leading modifiable contributors to dementia burden, with both ambient and household air pollution emerging as important environmental targets. Recently, neuropathological studies have shown that long-term exposure to fine particulate matter is associated with greater severity of Alzheimer's disease pathology,^25^ providing biological plausibility, while population-based cohorts consistently report that higher PM_2_._5_ exposure is linked to faster cognitive decline and increased dementia risk.^26^ Therefore, ambient particulate matter pollution may contribute to overall dementia burden through mechanisms not limited to disease occurrence alone. Taken together, these findings strengthen the rationale for coordinated prevention strategies, spanning air-quality improvement, cardiometabolic risk reduction, and broader social determinants such as education, that could substantially reduce the future dementia burden in Europe.

Recent regulatory approval of disease-targeted therapies for Alzheimer's disease creates new opportunities but also major planning challenges.^10^ Eligibility is restricted to individuals in the earliest symptomatic stages of Alzheimer's disease with biomarker-confirmed amyloid pathology, and treatment requires substantial diagnostic, monitoring, and specialist capacity.^10^^,^^15^^,^^27^^,^^28^ Furthermore, major gaps in case detection substantially limit the real-world impact of these therapies.^14^ Population ageing introduces an additional biological constraint. In most Western European countries, the majority of dementia cases occur in individuals aged over 80 years,^21^ in whom mixed neuropathology is common and clinicopathological correlations are often complex.^29^ Evidence from population-based and neuropathological studies has repeatedly shown that substantial pathological burden may be tolerated without overt clinical dementia during life, underscoring the limitations of single-mechanism therapeutic approaches in very old populations.^30^ In this setting, all-cause dementia estimates provide the population-level denominator needed to plan diagnostic capacity, biomarker assessment, specialist referral, treatment eligibility evaluation, monitoring, prevention strategies, and long-term care. Although subtype- and stage-specific data remain important for treatment-specific forecasting, their applicability may vary across age groups, particularly in very old populations in whom mixed neuropathology is common.^29^

This study has limitations. First, original epidemiological data were not available for all countries. Instead, GBD estimates are based on Bayesian meta-regression (DisMod-MR 2.1) to generate estimates in data-sparse settings. Although this approach enables internally consistent estimates across geographies, it assumes that prevalence can be inferred from regional patterns and covariates. Data availability for dementia epidemiology remains highly uneven across Europe; a comprehensive mapping of European cohort studies has shown that population-based data are heavily concentrated in Western Europe, with only very limited representation from Central and especially Eastern Europe, where only a small number of cohorts have been identified.^31^ This imbalance is also evident in the country-level data availability underlying the present GBD 2023 estimates, as illustrated in Appendix Figure S1. Our results, however, are broadly consistent with independent efforts such as the *Dementia in Europe Yearbook*,^8^ supporting their validity. Second, we focused on all-cause dementia because GBD does not provide internally consistent estimates for clinical subtypes. Though understanding the estimated numbers of individuals with Alzheimer's disease pathology is important given the new development in treatments, this is challenging given the high prevalence of mixed pathologies, the subsequent lack of alignment between clinical and pathological subtypes, and the lack of population-based data on pathologies, especially *in vivo*.^29^ Only a few meta-analyses have systematically synthesised such data,^32^^,^^33^ and important gaps remain in understanding their global impact. Third, risk-factor attribution is constrained by the limited set of exposures quantified in the current GBD framework, which does not fully capture the broader range of modifiable risks highlighted in recent Lancet Commissions.^11^ Furthermore, cross-country comparisons should also be interpreted cautiously, because observed differences may partly reflect variation in diagnostic ascertainment and death certification and coding practices. Death certificates are a particularly imperfect source for estimating dementia mortality because dementia may be omitted even when present, recorded only as a contributing condition, variably selected as the underlying cause, or coded differently across countries and over time. This is especially relevant because people living with dementia often die in the context of intercurrent or terminal events, such as pneumonia, infections, cardiovascular events, frailty-related complications, or other comorbid conditions, which may be preferentially recorded as the underlying cause of death. The extent of this problem is illustrated by cohort-based estimates from the United States, where dementia appears to contribute to many more deaths than are captured in official death-certificate statistics.^34^ Similarly, evidence from the UK Medical Research Council Cognitive Function and Ageing Studies in England and Wales showed that the accuracy of dementia recording on death certificates improved substantially over time, but remained incomplete and was influenced by dementia severity, institutional residence, and place of death.^35^ To address these challenges, the GBD does not rely directly on death certificate coding alone, but applies corrections based on an attributable-risk framework informed by excess mortality. Although this methodology avoids introducing bias from variable or incorrect cause of death coding, it requires strong assumptions and risks propagating uncertainty from prevalence estimates to mortality estimates.^36^ Finally, further investment in high-quality, population-based epidemiological studies, particularly in underrepresented regions, is essential to improve precision, track temporal trends in incidence and prevalence, and clarify regional differences in the distribution of dementia burden.

Dementia is one of the fastest-growing public health challenges in Europe, with nearly 8 million people affected in the EU-27 in 2023 and numbers projected to rise further as populations age. Although age-standardised prevalence has shown slight declines, the absolute impact, reflected in prevalence, mortality, and DALYs, continues to increase, with disproportionate effects on women and older adults. This growing impact underscores both the urgency of action and the potential for change. The trajectory of dementia can be shaped by preventive measures, including improvements in air quality, better control of cardiometabolic risks, and investments in education and other social determinants. In parallel, the emergence of disease-targeted therapies for early symptomatic Alzheimer's disease in Europe emphasises the need for robust, country-specific, and where possible subtype- and stage-specific, estimates to guide diagnostic capacity, equitable access, and sustainable planning within ageing health systems. These estimates also make clear that dementia surveillance in Europe must do more to capture underserved and socioeconomically disadvantaged populations, including groups who remain underdiagnosed or poorly represented in routine data systems. Addressing these gaps through dedicated studies will be essential to ensure that dementia research and policy are inclusive and consistent with the health equity objectives of WHO and the European Union. Integrating prevention, therapeutic innovation, and equitable long-term care within coherent strategies will be essential to mitigate the rising impact of dementia across Europe.

## Contributors

DU and GL contributed to the conception and design of the study. DU accessed the data, performed the analyses, and draughted the first version of the manuscript. DU and GL directly accessed and verified the underlying data. DU, EN, SG, CB, NR, and GL interpreted the findings and critically revised the manuscript for important intellectual content. DU and GL had final responsibility for the decision to submit the manuscript for publication. All authors approved the final version of the manuscript.

## Data sharing statement

All data used in this study are publicly available through the Global Health Data Exchange GBD 2023 website at https://vizhub.healthdata.org/gbd-results/.

## Editor note

The Lancet Group takes a neutral position with respect to territorial claims in published maps and text.

## Declaration of interests

Giancarlo Logroscino has served as an investigator in clinical trials sponsored by Biogen, Axovant, Alector, Denali, Roche, Eisai, Genentech, Amylyx, and PIAM Farmaceutici SpA, and has received consulting fees and honoraria for lectures from Eisai, Roche, Eli Lilly, PIAM Farmaceutici SpA, and Biogen. Emma Nichols reports grants or contracts from NIH/NIA, consulting fees from SHARE-ERIC and Columbia University, honoraria from NIH/NIA, and travel support from the University of Michigan. All other authors declare no competing interests.
