# Supplementary material for: Burden of Dementia in Europe, 1990–2023: a systematic analysis from the Global Burden of Disease Study 2023
Source: Lancet Reg Health Eur. 2026 Jul 31;68:101796. doi: 10.1016/j.lanepe.2026.101796 (PMC13452175; doi:10.1016/j.lanepe.2026.101796)
Supplement: Supplementary file 1 [file mmc1.docx]

**SUPPLEMENTARY MATERIAL TO**

**Burden of dementia in Europe, 1990-2023: a systematic analysis from the Global Burden of Disease Study 2023**

**Daniele Urso^1,2^ MD, MPH; Emma Nichols, PhD^3^; Stefano Giannoni-Luza^1,4^, MD, MPH; Carol Brayne^5^, MD; Nicolas Ray^2^, PhD; Giancarlo Logroscino^1,4^, MD, PhD**

**1 Center for Neurodegenerative Diseases and the Aging Brain, University of Bari 'Aldo Moro', "Pia Fondazione Cardinale G. Panico", Tricase, Lecce, Italy**

**2 GeoHealth group, Institute of Global Health, Faculty of Medicine, University of Geneva, Geneva, Switzerland**

**3 Center for Economic and Social Research, University of Southern California, Los Angeles, CA, USA**

**4 Department of Translational Biomedicine and Neuroscience (DiBraiN), University of Bari ‘Aldo Moro’, Bari, Italy**

**5 Cambridge Public Health, University of Cambridge, Cambridge, UK**

**TABLE OF CONTENT**

**Table S1. Checklist for Guidelines of Accurate and Transparent Health Estimates Reporting (GATHER)**

**Figure S1. Country-level data availability for age-standardised prevalence of dementia in the WHO European Region, 2023.**

**Figure S2.** Age-standardised prevalence rate (ASR) of dementia per 100,000 population in the WHO European Region, 2023

### **Figure S3.** Age-specific prevalence of dementia in the WHO European Region, 2023, by sex

**Figure S4. Rank changes in age-standardised death rates by cause, European Union (EU-27), both sexes, 1990 vs 2023**

**Figure S5. Rank changes in age-standardised death rates by cause, WHO European Region, both sexes, 1990 vs 2023**

**Figure S6. Decomposition of the change in dementia DALYs between 1990 and 2023 in the EU-27 and the WHO European Region, by population growth, population ageing, and changes in age-specific DALY rates.**

**Figure S7. Association between socio-demographic development (SDI) and age-standardised DALY rates for dementia across countries of the WHO European Region in 2023.**

**Figure S8. Association between socio-demographic development (SDI) and age-standardised DALY rates for dementia across EU-27 countries in 2023.**

**Checklist of information that should be included in new reports of global health estimates**

| **Item #** | **Checklist item** | **Reported on page #** |
| --- | --- | --- |
| **Objectives and funding** | | |
| **1** | Define the indicator(s), populations (including age, sex, and geographic entities), and  time period(s) for which estimates were made. | 5-8 |
| **2** | List the funding sources for the work. | 2 |
| **Data Inputs** | | |
| *For all data inputs from multiple sources that are synthesized as part of the study:* | | |
| **3** | Describe how the data were identified and how the data were accessed. | 7-10 |
| **4** | Specify the inclusion and exclusion criteria. Identify all ad‐hoc exclusions. | 7-9 |
| **5** | Provide information on all included data sources and their main characteristics. For each data source used, report reference information or contact name/institution, population represented, data collection method, year(s) of data collection, sex and age range,  diagnostic criteria or measurement method, and sample size, as relevant. | 9-10 and appendix Figure S1 |
| **6** | Identify and describe any categories of input data that have potentially important biases  (e.g., based on characteristics listed in item 5). | 9 |
| *For data inputs that contribute to the analysis but were not synthesized as part of the study:* | | |
| **7** | Describe and give sources for any other data inputs. | 10-12 |
| *For all data inputs:* | | |
| **8** | Provide all data inputs in a file format from which data can be efficiently extracted (e.g., a spreadsheet rather than a PDF), including all relevant meta‐data listed in item 5. For any data inputs that cannot be shared because of ethical or legal reasons, such as third‐party ownership, provide a contact name or the name of the institution that retains the right to  the data. | Data sharing statement (Page 26) and GHDx Sources Tool |
| **Data analysis** | | |
| **9** | Provide a conceptual overview of the data analysis method. A diagram may be helpful. | 7-12 |
| **10** | Provide a detailed description of all steps of the analysis, including mathematical formulae. This description should cover, as relevant, data cleaning, data pre‐processing, data adjustments and weighting of data sources, and mathematical or statistical  model(s). | 7-14 |
| **11** | Describe how candidate models were evaluated and how the final model(s) were  selected. | 7–12 and GBD 2023 capstone references (refs 16–17) |
| **12** | Provide the results of an evaluation of model performance, if done, as well as the results  of any relevant sensitivity analysis. | GBD 2023 Disease and Injury and Risk Factor Collaborators (ref 17) |
| **13** | Describe methods for calculating uncertainty of the estimates. State which sources of  uncertainty were, and were not, accounted for in the uncertainty analysis. | 13 |
| **14** | State how analytic or statistical source code used to generate estimates can be accessed. | 14 |
| **Results and Discussion** | | |
| **15** | Provide published estimates in a file format from which data can be efficiently extracted. | Data sharing statement; GBD Results Tool (vizhub.healthdata.org) |
| **16** | Report a quantitative measure of the uncertainty of the estimates (e.g. uncertainty  intervals). | 15-20 |
| **17** | Interpret results in light of existing evidence. If updating a previous set of estimates,  describe the reasons for changes in estimates. | 21-24 |
| **18** | Discuss limitations of the estimates. Include a discussion of any modelling assumptions or  data limitations that affect interpretation of the estimates. | 24-26 |

This checklist should be used in conjunction with the GATHER statement and Explanation and Elaboration document, found on gather‐statement.org

**Figure S1. Country-level data availability for age-standardised prevalence of dementia in the WHO European Region, 2023.**

Colours represent the number of country-level contributions identified in the extracted sources, with darker shades indicating greater availability. Grey indicates no identified country-level contribution.

**Figure S2. Age-standardised prevalence rate (ASR) of dementia per 100,000 population in the WHO European Region, 2023**

### **Figure S3. Age-specific prevalence of dementia in the WHO European Region, 2023, by sex**

### Prevalence is expressed as the percentage of the population affected within each 5-year age group. Bars represent point estimates for females and males, with error bars indicating 95% uncertainty intervals.

**Figure S4. Rank changes in age-standardised death rates by cause, European Union (EU-27), both sexes, 1990 vs 2023**

Slopegraph showing the leading causes ordered by age-standardised deaths per 100 000. Lines connect ranks in 1990 (left) to 2023 (right). Colours denote GBD cause groups: communicable, maternal, neonatal, and nutritional diseases (orange), non-communicable diseases (blue), and injuries (green).

**Figure S5. Rank changes in age-standardised death rates by cause, WHO European Region, both sexes, 1990 vs 2023**

Slope-graph showing the leading causes ordered by age-standardised deaths per 100 000. Lines connect ranks in 1990 (left) to 2023 (right). Colours denote GBD cause groups: communicable, maternal, neonatal, and nutritional diseases (orange), non-communicable diseases (blue), and injuries (green).

**Figure S6. Decomposition of the change in dementia DALYs between 1990 and 2023 in the EU-27 and the WHO European Region, by population growth, population ageing, and changes in age-specific DALY rates.**

Bars show the proportional contribution of population growth, population ageing, and changes in age-specific DALY rates to the overall increase in total dementia DALYs between 1990 and 2023. Positive values indicate factors contributing to an increase in DALYs, whereas negative values indicate factors offsetting this increase.

**Figure S7. Association between socio-demographic development (SDI) and age-standardised DALY rates for dementia across countries of the WHO European Region in 2023.**

Each point represents one country in the WHO European Region. The x-axis shows the Socio-demographic Index (SDI), a composite measure of income per capita, educational attainment, and fertility, and the y-axis shows the age-standardised DALY rate for dementia per 100 000 population in 2023. The fitted line illustrates the overall positive association between SDI and dementia DALY rates.

**Figure S8. Association between socio-demographic development (SDI) and age-standardised DALY rates for dementia across EU-27 countries in 2023.**

Each point represents one EU-27 country. The x-axis shows the Socio-demographic Index (SDI), a composite measure of income per capita, educational attainment, and fertility, and the y-axis shows the age-standardised DALY rate for dementia per 100 000 population in 2023. The fitted line illustrates the overall positive association between SDI and dementia DALY rates.
